# A Personal Historical Perspective on Psychiatry in Japan During the Last 4 Decades

**DOI:** 10.14789/jmj.JMJ23-0021-R

**Published:** 2023-08-25

**Authors:** TOSHIHITO SUZUKI

**Affiliations:** 1 Department of Psychiatry, Juntendo University Koshigaya Hospital, Saitama, Japan; 1 Department of Psychiatry, Juntendo University Koshigaya Hospital, Saitama, Japan; 2 Department of Psychiatry & Behavioral Science, Juntendo University Graduate School of Medicine, Tokyo, Japan; 2 Department of Psychiatry & Behavioral Science, Juntendo University Graduate School of Medicine, Tokyo, Japan

**Keywords:** animal models of schizophrenia, Juntendo University Koshigaya Hospital, perinatal mental health, development of guidelines

## Abstract

After graduating from University of Tsukuba in 1982, I joined the Department of Psychiatry at the same university. Due to the anti-psychiatry social movement and reports of incidents involving violence against in-hospital patients at psychiatric hospitals, psychiatric associations in Japan faced questions related to ethical awareness, making it a challenging environment for conducting clinical research. For this reason, the first half of my journey─my 20 years at the University of Tsukuba─was spent conducting basic research on animal models of schizophrenia. With respect to the onset of schizophrenia, I studied dopamine and related neuropeptides in the brain, as well as abnormalities in neurotransmission in the excitatory and inhibitory amino acid neurotransmission systems.

In April 2002, I was appointed as a Department Chair at Juntendo University Koshigaya Hospital. I was responsible for overseeing many medical staff, including the clinical education of practicum students and resident physicians, as well as the training of psychiatric specialists. I was also involved in the management and operation of medical services provided at the mental health clinic that had 350 outpatients per day and saw the admission and discharge of 500 patients annually. Meanwhile, I became actively involved in activities related to perinatal mental health. In 2018, I was appointed as the Director of the Japanese Society of Perinatal Mental Health and worked diligently to improve medical care related to perinatal mental health in Japan through the development of perinatal mental health guidelines.

## Introduction

On the occasion of my retirement, I would like to impart some insights on my 40-year journey thus far and provide an overview of the transition that took place in the field of psychiatry in Japan during this period. After graduating from the School of Medicine, University of Tsukuba in 1982, I joined the Department of Psychiatry (chaired by Professor Junzo Koizumi) at the same university. Upon returning from studying abroad at the Department of Pharmacology and Toxicology at the University of Mississippi Medical Center (chaired by Professor Ing Kang Ho), I became an Associate Professor at the Department of Psychiatry, University of Tsukuba. In April 2002, under the Chief Professor, Dr. Heii Arai, I was appointed as a Department Chair at Juntendo University Koshigaya Hospital. In 2008, I became a professor (intradepartmental) at the Department of Psychiatry of Juntendo University Koshigaya Hospital which then led to my appointment as the Hospital Director at this hospital in April 2021, a position I still hold today.

## Basic research and clinical activities during my years at the University of Tsukuba

In the early 1980s, when I first started at the Department of Psychiatry, University of Tsukuba, Japan's psychiatric associations were experiencing the rippling effects of the anti-psychiatry social movement. The Japanese Society of Psychiatry and Neurology suspected several domestic clinical studies of ethical violations. Furthermore, even in the field of psychiatric care, reports of violent incidents perpetrated by mental health providers against in-hospital patients were surfacing. It was a time when these incidents concerning human rights issues involving psychiatric patients were being condemned. Associations in biological psychiatry, which developed into Japanese Society of Biological Psychiatry later, were forced to cancel their academic conferences in some years. This is why the Japanese Society of Psychiatry and Neurology established the “Committee on Research and Human Rights” in 1984 to monitor clinical research ethics. However, these were days during which the understanding with regard to research ethics in psychiatry was still undeveloped, and it was a challenging environment for conducting psychiatric clinical research. This historical backdrop significantly impacted the direction of my subsequent research life.

## Research of abnormal neurotransmission systems in the brain in animal models of schizophrenia

After joining the department, I devoted myself to basic research, conducting animal experiments on schizophrenia models. Through repeated administrations of psychostimulants such as methamphetamine (MAP) to rats, we created rats that exhibited reverse tolerance or behavioral sensitization─the animal model of schizophrenia. My doctoral thesis focused on the cholecystokinin (CCK) system─a neuropeptide system that regulates dopaminergic neuronal systems─and I reported about the receptor abnormalities in the frontal cortex of the same nervous system.

**Figure 1:**
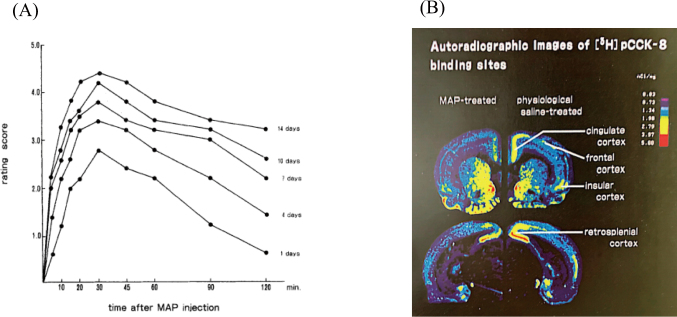
(A) The rating score after methamphetamine injection at every test session. The behavioral changes were manifested much more rapidly and the mean peak values were increased with the number of injections. It means behavioral sensitization or reverse tolerance (cited from ref. 1). (B) Autoradiographic images of [^3^H]pCCK-8 binding sites in the rat brain. Brain section images of physiologic saline-treated rats (on the right side) and those of methamphetamine-treated rats (on the left side) are shown (cited from ref. 1).

In 1989, using the in vitro quantitative receptor autoradiographical technique, changes in the binding parameters of [propionyl-^3^H] propionylated CCK-8 ([^3^H]pCCK-8) binding sites in the rat forebrain were investigated following acute and chronic administration (14 days) of MAP (4mg/kg/day)^1)^. The (Kd)app values of [^3^H]pCCK-8 binding sites in the frontal medial cortex and anterior cingulate cortex were significantly reduced after a single injection of 4 mg/kg MAP. On the other hand, chronic treatment with MAP at this dose significantly decreased the B_max_ value of [^3^H]pCCK-8 binding sites in the anterior cingulate cortex accompanied by supersensitivity of locomotor effects to MAP (Figure 1 (A), (B)). These findings suggest that dopamine neurons in these two regions are functionally related to intrinsic CCK-containing cortical neurons, and that CCK subsensitivity, perhaps due to an alteration in DA transmission, is involved in MAP sensitization. These findings may be relevant to the DA hypothesis of schizophrenia.

While studying abroad in the United States (1995 to 1996) at the Department of Pharmacology and Toxicology, the University of Mississippi Medical Center, I researched the changes in gamma-aminobutyric acid (GABA)-benzodiazepine receptors in barbiturate dependence. During this process, I mastered the in-situ hybridization method, which led to my subsequent research on the role of receptor mRNA subunits.

In 1995, changes in benzodiazepine binding sites labeled by [^3^H]flunitrazepam (FNZ) in twenty discrete brain regions of rats made tolerant to and dependent upon pentobarbital were examined^2)^. Animals were rendered tolerant by intracerebroventricular infusion with pentobarbital (300 micrograms/ 10 microliters/hr for six days) through pre-implanted cannulae connected to osmotic mini-pumps. The pentobarbital dependence was assessed 24 hr after abrupt withdrawal from pentobarbital. In the tolerant rats, a significant increase in [^3^H]FNZ binding sites was found in the frontal cortex and the molecular layer of olfactory bulb. [^3^H]FNZ binding sites in the pentobarbital dependent rats were significantly increased in the frontal cortex, caudate-putamen, olfactory tubercle, globus pallidus and ventral pallidum, in addition to those observed in the tolerant group. Taken together with characteristics of subtypes of benzodiazepine receptors and changes in GABA-benzodiazepine receptor complexes elucidated in previous studies, these findings suggest that both types of benzodiazepine receptors are involved in the development of pentobarbital intoxication mediated by GABA_A_ receptors.

Upon returning to Japan, I conducted research related to receptor mRNA subunits of excitatory (glutamatergic) and inhibitory (GABAergic) amino acid neurotransmission systems in the brain of animal models of schizophrenia. At the time, the amino acid neural transmission was a research area of increased focus since antipsychotics, which are dopamine receptor blockers in drug therapy for schizophrenia, were not effective among some patients with positive symptoms and also lacked effectiveness against negative symptoms themselves.

**Figure 2:**
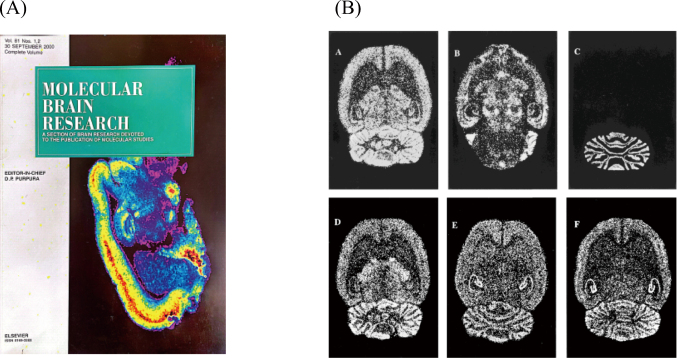
(A)Autoradiographic images labeled by [^35^S]TBPS in rat brain published on the cover of the journal “Molecular Brain Research” (cited from ref. 3). (B)The distributions of mRNAs of each of α1 (upper portion) (A), α2 (lower portion) (B), α6 (C), β2 (D), β3 (E), and γ2 (F) subunits. Each mRNA subunit shows a differential distribution (cited from ref. 3).

In 2000, the effects of intermittent intraperitoneal (i.p.) administration of cocaine (20 mg/kg/day) on GABA_A_-benzodiazepine (BZD) receptors labeled by t-[^35^S]butylbicyclophosphorothionate (TBPS), and on several types of mRNA subunits were investigated in rat brain by in vitro quantitative receptor autoradiography (Figure 2 (A)) and in situ hybridization (Figure 2 (B))^3)^. There was a significant decrease in the level of *α*1, *α*6, *β*2, *β*3, and *γ*2 subunits mRNA, with no alteration of [^35^S]TBPS binding in any regions in the brain of rats at 1 h following a single injection of cocaine. In chronically treated animals, the mean scores of stereotyped behavior were increased with the number of injections. The level of *β*3 subunit mRNA was decreased in the cortices and caudate putamen, at 24 h after a final injection of chronic administrations for 14 days. These findings suggest that the disruption of GABA_A_-BZD receptor formation is closely involved in the development of cocaine-related behavioral disturbances. Further studies on the physiological functions on GABA_A_-BZD receptor complex will be necessary for an explanation of the precise mechanisms underlying the development of hypersensitization of cocaine.

**Figure 3:**
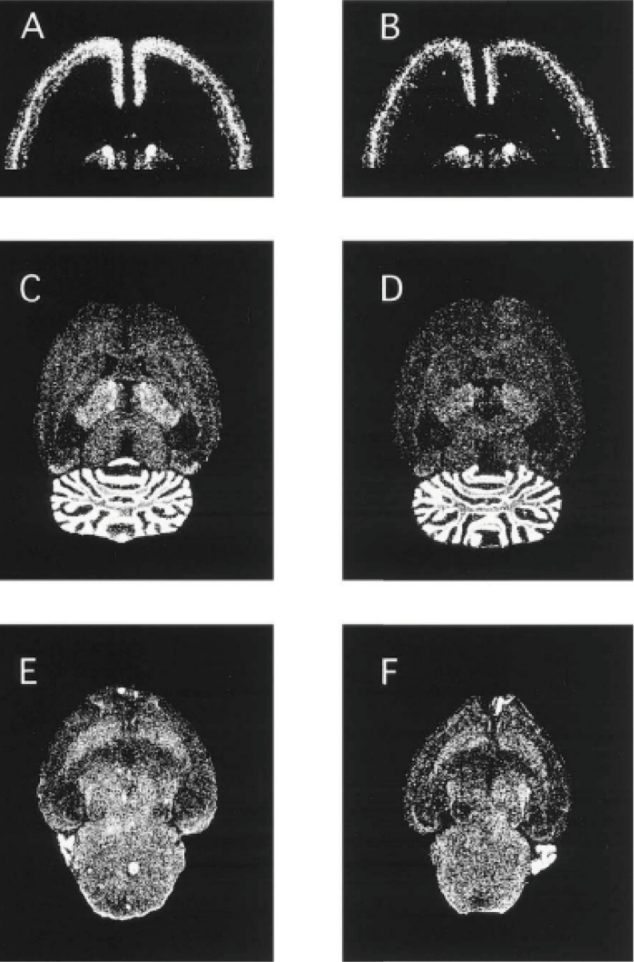
The autoradiograms of mGluR mRNA in horizontal upper and lower sections in rat brain after repeated administration. The left side (A, C, E) is treated with saline and the right side (B, D, F) is treated with phencyclidine (cited from ref. 4).

In 2001, the following animal studies regarding phencyclidine (PCP), which induces psychotic symptoms in humans, have suggested that metabotropic glutamate receptors (mGluRs) represent a novel target for the treatment of PCP psychosis. We used in situ hybridization to investigate the gene expressions of the mGluR 1-5 subtypes following repeated administration of PCP in rats^4)^. After repeated PCP administration for 14 days, the mGluR2 mRNA expression of group II mGluR in the anterior cingulate cortex and the mGluR4 mRNA expression of group III mGluR in the cortical regions, the caudate putamen, thalamus, and subiculum were significantly decreased (Figure 3). These results indicate that it is of particular interest that mGluR2 and mGluR4subtype is involved in a development of behavioral abnormality following repeated PCP administration.

In 2002, we subsequently investigated the effects of i.p. injections of cocaine (20 mg/kg/day) on subunit mRNAs of *N*-methyl-D-aspartate (NMDA) receptors (NR1/NR2A-2C) in the rat brain by in situ hybridization using phosphor screen analysis^5)^. The level of NR1 subunit mRNA significantly increased in hippocampal complexes 1 h after a single i.p. injection of cocaine. After repeated cocaine injection, the mean scores of stereotyped behaviour were increased with the number of injections. The level of NR1 subunit mRNA was obviously decreased in the striatum and cortices 24 h after a final injection following 14 days of subchronic administration. Levels of NR2B subunit mRNA were reduced in the cortices and striatum. These findings suggest that the disruption of NR1 and NR2B subunits in the discrete brain regions occurs under the cocaine-related behavioral abnormalities and would be closely implicated in the initiation and expression of behavioral sensitization induced by repeated cocaine administration.

## A memorable clinical report

In the 1980s in Japan, case reports were more actively reported than large-scale clinical studies, and borderline personality disorder and eating disorders were attracting much attention. A personally memorable case for me was a patient who had long been diagnosed with schizophrenia. The results of a detailed examination revealed that this patient had mitochondrial encephalomyopathy, and this case appeared as a case report in an international journal below.

**Figure 4:**
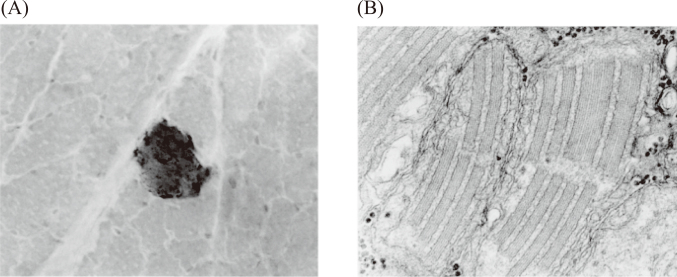
(A) Ragged red fibers with red granular material in the subsarcolemmal region from biopsied muscle tissue of the quadriceps. Modified Gomori-trichrome stain (cited from ref. 6). (B) Electron microscopic examination revealed enlargement of mitochondria and aggregation of abnormal mitochondria with paracrystalline formation (x63000) (cited from ref. 6).

In 1989, the author reported a case of a 37- year-old man with mitochondrial encephalomyopathy^6)^. The recurrent mental disorder with auditory hallucination, delusion of reference was the main remarkable symptom during the earlier stage of illness. These symptoms were regarded as a schizophrenia-like symptoms. The present case was finally diagnosed as mitochondrial encephalomyopathy with schizophrenia-like symptoms. Ragged-red fibers and crystalline inclusions in mitochondria were revealed by biopsy of the striated muscle of the patient (Figure 4 (A), (B)). Mitochondrial myopathy, encephalopathy, lactic acidosis and stroke-like episodes (MELAS) was diagnosed clinically. In addition to severe atrophy and degeneration of the generalized striated muscles, many foci of laminar necrosis of the cerebral cortex and the abnormalities of general organs were observed. We suggested that mitochondrial encephalomyopathy can cause the organic brain syndromes showing schizophrenia-like mental symptoms.

## Clinical activities and education at the Juntendo University Koshigaya Hospital and transitions in psychiatry

**Figure 5:**
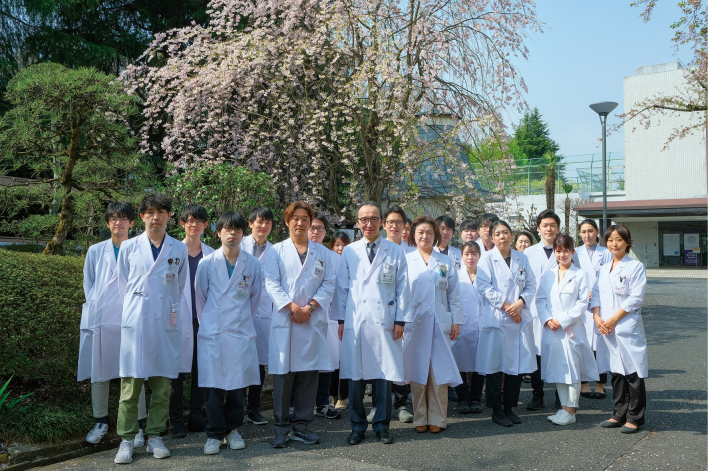
Medical staff in the Department of Psychiatry, Juntendo Koshigaya Hospital

I joined the Juntendo University Koshigaya Hospital in April 2002, and for 21 years until my retirement, I served as a Department Chair and endeavored to enhance clinical activities and ensure medical safety at our hospital's mental clinic. Being a 226-inpatient-bed psychiatric hospital, we were committed to offering clinical education for the 5^th^ grade medical students under the on site training and resident physicians and assisting young physicians aspiring to become psychiatrists in obtaining their qualifications as specialists and designated physicians (Figure 5). In the 2000s, a new trend swept across the field of psychiatry. In the field of psychiatric care, the concept of dementia started to gain attention. The Japanese name of the illness changed from “chihosho”, which is a pejorative word for dementia, to “ninchisho”, which is a descriptive word for neurocognitive disorder, and the therapeutic drugs for schizophrenia and depression transitioned from first-generation to second-generation therapeutic drugs. Further, in daily clinical practice, a decline in adverse events related to antipsychotics and antidepressants dramatically improved medication adherence, leading to more proactively conducting clinical studies on schizophrenia and depression than before. Our hospital also reported the results of a clinical study on depression. Less attention has been given to sex differences in the underlying biological mechanisms of depression. The adrenal androgens, dehydroepiandrosterone (DHEA) and its sulfate derivative (DHEA-S), play a critical role in controlling affect, mood, and anxiety.

In 2013, a clinical study examining the role of male sex hormones in depression is presented. The objective of the present study was to investigate differences in serum levels of adrenal androgens in male and female patients with major depressive disorder (MDD)^7)^. Participants included 90 inpatients with MDD at the psychiatric ward of Juntendo University Koshigaya Hospital who were receiving antidepressants. Matched controls included 128 healthy individuals. Serum DHEA levels were significantly increased in both male and female MDD patients compared with controls. Serum levels of DHEA-S in male patients were significantly decreased compared with male controls. No significant correlations were seen between adrenal androgens and HAM-D scores in male or female patients. Multiple regression analysis showed that both hormones were affected by the age at onset of depression. Elevated levels of serum DHEA may be associated with the biological pathophysiology of depression, as DHEA administration has been found to be effective for the treatment of depression.

## Contribution to perinatal mental health in Japan

**Figure 6:**
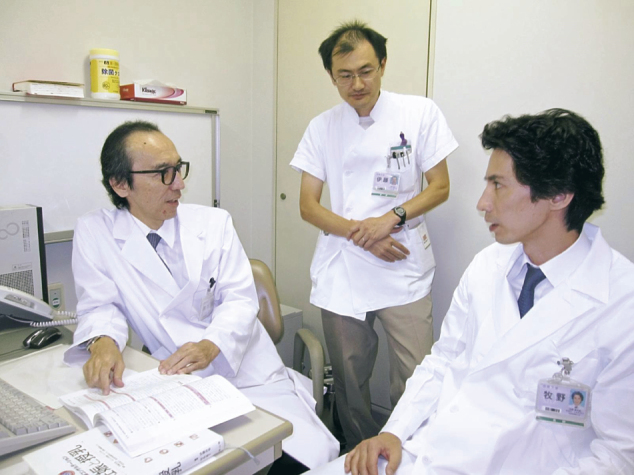
An outpatient clinic specializing in perinatal mental health was featured in the mass media. The photo (Oct. 27, 2014) is reprinted by courtesy of THE YOMIURI SHIMBUN.

**Figure 7:**
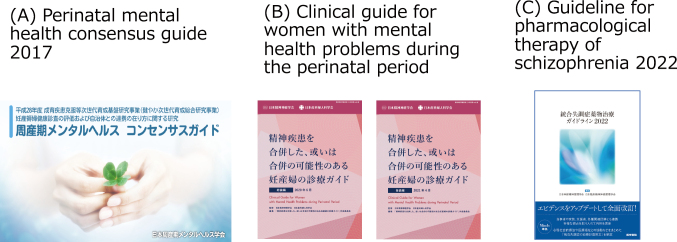
(A) Perinatal mental health consensus guide 2017. The Japanese Society of Perinatal Mental Health, the Japan Association of Obstetricians and Gynecologists and the Japan Society of Obstetrics and Gynecology collaborated on this project. (B) Clinical guide for women with mental health problems during the perinatal period. The guideline was created by cooperation of the following two fields, the Japanese Society of Psychiatry and Neurology and the Japan Society of Obstetrics and Gynecology. Overview in May 2020, and its Detailed Contexts in April 2021 have been reported, respectively. (C) Guideline for pharmacological therapy of schizophrenia 2022. The guideline was created by the Japanese Society of Neuropsychopharmacology and the Japanese Society of Clinical Neuropsychopharmacology.

In the field of psychiatry, and among the various areas therein, after 2010, a concept referred to as “unmet needs” began to draw attention. One of these areas was perinatal mental health in the psychiatry. In 2014, Juntendo University Hospital established an outpatient clinic specializing in perinatal mental health. At the time, this drew attention and was featured in the mass media (Figure 6). I was appointed as the Director of the Japanese Society of Perinatal Mental Health in 2018, and I have contributed many reviews in domestic journals about the challenges to and prospects for improving perinatal mental health in Japan. In 2017, as the Deputy Chairperson, I was involved in creating the Consensus Guidelines on Perinatal Mental Health 2017 (Figure 7 (A)). Through a joint collaboration between the Japanese Society of Psychiatry and Neurology and the Japan Society of Obstetrics and Gynecology, a committee was then formed to develop perinatal mental health guidelines. I served as the Chairperson of the Guidelines Preparation Committee, and the committee developed the overview in 2020^8)^ and subsequently the detailed contents in 2021^9)^(Figure 7 (B)). Recently, Guideline for pharmacological therapy of schizophrenia 2022 was created by the Japanese Society of Neuropsychopharmacology and the Japanese Society of Clinical Neuropsychopharmacology. (Figure 7 (C)). I acted as an adviser in the field of perinatal mental health of schizophrenia.

Keeping in mind the limitations of animal disease models in the field of perinatal mental health, basic research was conducted with a focus on clinical conditions of perinatal mental health. In pregnant women with epilepsy, it is imperative to balance the safety of the mother and the potential teratogenicity of anticonvulsants, which could cause impairments such as intellectual disability and cleft lip.

**Figure 8:**
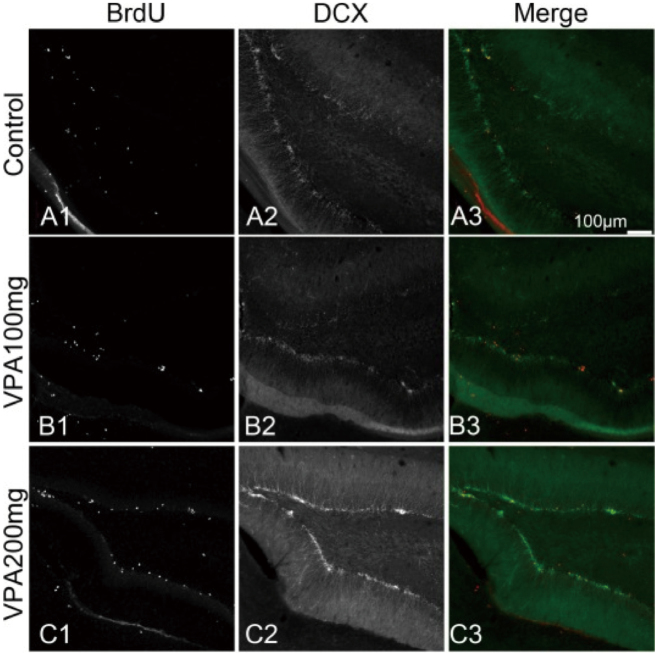
Hippocampal cell differentiation with BrdU and doublecortin(DCX) hippocampal dentate gyrus sections double immunostained for BrdU and DCX was shown (cited from ref. 10).

In 2019, we examined behavioral and hippocampal neurogenesis alterations in male offspring of rats exposed to valproic acid (VPA) during pregnancy^10)^. Pregnant Wistar rats received daily intraperitoneal injections of VPA (100 mg/kg/day or 200 mg/kg/day) from embryonic day 12.5 until birth. At postnatal day 29, animals received an injection of bromodeoxyuridine (BrdU). At postnatal day 30, animals underwent the open field (OF), elevated plus-maze, and Y-maze tests. Of the offspring of the VPA200 mothers, 66.6% showed a malformation. In the OF test, these animals showed locomotor hyperactivity. In the elevated plus-maze, offspring of VPA-treated mothers spent significantly more time in the open arms. The number of BrdU-positive cells in the dentate gyrus of the offspring of VPA-treated mothers increased significantly in a dose-dependent manner compared with the control (Figure 8). In conclusion, VPA administration during pregnancy results in malformations and attention-deficit/hyperactivity disorder-like behavioral abnormalities in the offspring. Repeated use of high doses of VPA during pregnancy may increase the risk of neurodevelopmental abnormalities dose dependently and should be carefully considered.

## Conclusion

My 40 years as a psychiatrist were split into two phases: the first 20 years at the University of Tsukuba and the second 20 years at Juntendo University. Basic research was the focus during my days at the University of Tsukuba, while my activities at Juntendo University were concentrated on clinical research, education, and medical practice. I believe that the transition that I experienced as a psychiatrist reflects the changes of a psychiatrist who aspired to work in the field of biological psychiatry.

In April 2021, I was appointed as the Hospital Director at Juntendo University Koshigaya Hospital, where I have since been engaged in realizing a restructuring project. I plan to restructure the Juntendo University Koshigaya Hospital, a psychiatric hospital, into a general internal medicine hospital. From now on, I intend to focus my time on restructuring the Juntendo University Koshigaya Hospital from a psychiatric hospital into a new semi-general internal medicine hospital. I am committed to continue contributing to this development to the best of my capacity and aim to create a branding that sets us apart from other affiliated hospitals.

## Funding

No funding was received.

## Author contributions

TS contributed to the conception, drafting the manuscript, and preparation of figures.

## Conflicts of interest statement

The author has no conflict of interest to disclose.

## References

[1] Suzuki T, Moroji T: Cholecystokinin binding sites in the rat forebrain: effects of acute and chronic methamphetamine administration. J Neural Transm, 1989; 77: 181-195. 10.1007/BF01248931 2760604

[2] Suzuki T, Ito T, Wellman SE, Ho IK: Change in [^3^H]flunitrazepam binding in the rats made tolerant to and dependent upon pentobarbital. Life Sci, 1995; 57: PL63-69. 10.1016/0024-3205(95)00286-f 7623607

[3] Suzuki T, Abe S, Yamaguchi M, et al: Effects of cocaine administration on receptor binding and subunits mRNA of GABA_A_-Benzodiazepine receptor complexes. Synapse, 2000; 38: 198-215. 10.1002/1098-2396(200011)38:2<198::AID-SYN11>3.0.CO;2-K 11018794

[4] Abe S, Suzuki T, Ito T, et al: Effects of single and repeated phencyclidine administration on the expression of metabotropic glutamate receptor subtype mRNA in rat brain. Neuropsychopharmacology, 2001; 25: 173-184. 10.1016/S0893-133X(00)00250-5 11425501

[5] Yamaguchi M, Suzuki T, Abe S, et al: Repeated cocaine administration differentially affects NMDA receptor subunit (NR1, NR2A-C)mRNAs in rat brain. Synapse, 2002; 46: 157-169. 10.1002/syn.10132 12325043

[6] Suzuki T, Koizumi J, Shiraishi H, et al: Psychiatric disturbance in mitochondrial encephalopathy. J Neurol Neurosurg Psychiatry, 1989; 52: 920-922. 10.1136/jnnp.52.7.920-a PMC1031953 2769294

[7] Kurita H, Maeshima H, Kida S, et al: Serum dehydroepiandrosterone (DHEA) and DHEA-sulfate(S) levels in medicated patients with major depressive disorder compared with controls. J Affect Disord, 2013; 146: 205-212. 10.1016/j.jad.2012.09.004 23102506

[8] The Japanese Society of Psychiatry and Neurology and the Japan Society of Obstetrics and Gynecology: Clinical guide for women with mental health problems during the perinatal period: Overview. https://www.jspn.or.jp/uploads/uploads/files/activity/Clinical_guide_for_women_with_mental_health_problems_during_perinatal_period_ver1.2.pdf (Accessed May 31, 2023) (in Japanese)

[9] The Japanese Society of Psychiatry and Neurology and the Japan Society of Obstetrics and Gynecology: Clinical guide for women with mental health problems during the perinatal period: Detailed Contents. https://www.jspn.or.jp/uploads/uploads/files/activity/Clinical_guide_for_women_with_mental_health_problems_during_perinatal_period_details_ver1.2.pdf (Accessed May 31, 2023) (in Japanese)

[10] Kinjo T, Ito M, Seki T, et al: Prenatal exposure to valproic acid is associated with altered neurocognitive function and neurogenesis in the dentate gyrus of male offspring rats. Brain Res, 2019; 1723: 146403. 10.1016/j.brainres.2019.146403 31446017

